# Editorial: Advances in the understanding, diagnosis, and management of intracranial and extracranial arterial dissections

**DOI:** 10.3389/fneur.2026.1768064

**Published:** 2026-04-30

**Authors:** Timo Krings

**Affiliations:** 1 Neurointerventional Center, Chair, Division of Neurointerventional Radiology, UMass-Chan Lahey Department of Radiology, Lahey Hospital and Medical Center - Beth Israel Lahey Health, Boston, MA, United States; 2 UMass-Chan Lahey Department of Radiology, TH Chan School of Medicine, UMass Chan Medical School, Worcester, MA, United States; 3 Edward B. Singleton Department of Radiology, Texas Children's Hospital, Baylor College of Medicine, Houston, TX, United States; 4 Department of Medical Imaging, University of Toronto, Toronto, ON, Canada

**Keywords:** arterial dissection, histopathology, management, outcome, revascularization

Intracranial and extracranial arterial dissections represent a complex and clinically significant spectrum of cerebrovascular disease, particularly affecting young and middle-aged populations. These conditions are a well-recognized cause of ischemic stroke, subarachnoid hemorrhage, and other catastrophic neurological outcomes, yet they often remain diagnostically elusive because of their heterogeneous presentations and frequent overlap with other vascular and systemic disorders. Delays in recognition and treatment continue to pose substantial challenges to improving outcomes.

This Research Topic of *Frontiers in Neurology*, entitled “*Advances in the understanding, diagnosis, and management of intracranial and extracranial arterial dissections*,” was conceived to address these challenges by bringing together contemporary research that spans basic mechanisms, imaging innovations, clinical prediction, and evolving medical, endovascular, and surgical therapies. This Research Topic was coedited by Kaijun Zhao (Tongji University), Jianmin Liu (Naval Medical University, Shanghai), Shane Gao (Tongji University), and Wenyuan Zhao (Wuhan University), and Timo Krings (Lahey Hospital and University of Masachusetts). This collaboration between North America and China exemplifies the growing and productive partnership between leading Chinese and U.S. neurovascular centers, uniting complementary expertise, patient populations, and research perspectives to advance our field.

Several contributions in this Research Topic deepen our understanding of the biological underpinnings of arterial dissection. Cai et al. provide a quantitative histopathologic analysis of thrombi retrieved from patients with dissection-related acute ischemic stroke. Their findings demonstrate that, despite important clinical distinctions—such as younger age and fewer vascular risk factors—thrombus composition alone does not reliably differentiate arterial dissection from other stroke etiologies. This work underscores the multifactorial nature of dissection-related thrombogenesis and highlights the limitations of relying solely on thrombus histology for etiological classification.

Complementing these mechanistic insights, Lin et al. explore metabolic risk factors in spontaneous cervical artery dissection among young Asian patients. Their demonstration that mildly elevated plasma homocysteine levels are significantly associated with dissection-related ischemic stroke adds an important dimension to risk stratification and raises the possibility of targeted screening and preventive strategies in susceptible populations.

Imaging remains central to the diagnosis and longitudinal management of arterial dissections, and multiple articles in this Research Topic address this evolving domain. Shao and Wang provide a comprehensive review of high-resolution magnetic resonance vessel wall imaging (MR-VWI) in extracranial cervical artery dissection. They highlight the value of MR-VWI in detecting intramural hematoma, assessing lesion activity, and stratifying recurrence risk, thereby supporting more individualized treatment strategies.

Li et al. focus on carotid artery dissection recanalization, systematically reviewing imaging modalities and clinical factors that influence vascular healing. Their synthesis emphasizes the need for standardized imaging follow-up and underscores the current gaps in consensus-driven management due to the lack of large randomized trials.

Xu et al. advance this concept further by developing and validating a nomogram integrating ultrasonographic features and clinical variables to predict recanalization in extracranial internal carotid artery dissection. With strong discriminatory performance, this tool represents a step toward precision medicine in the management of dissection-related stroke.

The heterogeneity and unpredictability of arterial dissections are illustrated by several case-based and review articles. Yao et al. offer a thorough review of middle cerebral artery dissection, summarizing current knowledge on pathophysiology, imaging characteristics, and therapeutic options for this rare but increasingly recognized entity.

Kee et al. describe ruptured isolated spinal artery aneurysms as a rare manifestation of dissecting disease, combining illustrative cases with a focused literature review. Their work expands the recognized phenotypic spectrum of arterial dissection and highlights the need for individualized management strategies in uncommon presentations.

Mohasin and Krings present a compelling longitudinal case of an intracranial vertebral artery dissecting aneurysm that demonstrated initial growth followed by spontaneous regression under conservative management. This report challenges traditional assumptions regarding the uniformly aggressive nature of such lesions and reinforces the importance of careful patient selection and long-term imaging surveillance.

Management strategies for arterial dissection continue to evolve, particularly in the realms of thrombolysis and endovascular intervention. Wang et al. address the controversial role of intravenous thrombolysis in acute ischemic stroke secondary to intracranial vertebrobasilar artery dissection. Their findings suggest that, in carefully selected patients, thrombolysis can be safe and may be associated with favorable functional outcomes, supporting its consideration in clinical practice.

Duan et al. evaluate the use of the Tubridge flow diverter for dissecting aneurysms of the middle cerebral artery, demonstrating acceptable safety and efficacy even in distal vessel segments. Their analysis also identifies anatomical factors, such as strong branch involvement, that influence aneurysm healing and angiographic outcomes.

Finally, Shi and Yu provide a comprehensive review of the challenges and future prospects of endovascular treatment for spontaneous intracranial artery dissection. Drawing on both literature and institutional experience, they outline indications, technical considerations, and complication profiles, offering practical guidance for individualized decision-making.

Beyond its scientific contributions, this Research Topic reflects the strength of international collaboration in neurovascular research. The close partnership between institutions in China—including Tongji University, Naval Medical University, and Wuhan University—and U.S. centers such as Lahey Hospital & Medical Center, University of Massachusetts, Boston, has enabled the integration of diverse clinical experiences and methodological approaches. Such collaboration is essential for addressing rare but devastating conditions like arterial dissection, where multicenter data, shared expertise, and cross-cultural exchange are critical to progress.

Collectively, the articles in this Research Topic provide a comprehensive and forward-looking perspective on intracranial and extracranial arterial dissections. They illuminate advances in pathophysiological understanding, refine diagnostic and prognostic tools, and explore emerging therapeutic strategies. We hope this body of work will stimulate further collaborative research, inform clinical practice, and ultimately contribute to improved outcomes for patients affected by these challenging vascular disorders.

